# Modeling Network Public Opinion Propagation with the Consideration of Individual Emotions

**DOI:** 10.3390/ijerph17186681

**Published:** 2020-09-14

**Authors:** Peihua Fu, Bailu Jing, Tinggui Chen, Jianjun Yang, Guodong Cong

**Affiliations:** 1 School of Management and E-Business, Zhejiang Gongshang University, Hangzhou 310018, China; fph@mail.zjgsu.edu.cn (P.F.); jingblue9735@163.com (B.J.); 2 School of Statistics and Mathematics, Zhejiang Gongshang University, Hangzhou 310018, China; 3 Department of Computer Science and Information Systems, University of North Georgia, Oakwood, GA 30566, USA; Jianjun.Yang@ung.edu; 4 School of Tourism and Urban-Rural Planning, Zhejiang Gongshang University, Hangzhou 310018, China; cgd@mail.zjgsu.edu.cn

**Keywords:** individual emotion, online public opinion, public opinion propagation, viewpoint interaction

## Abstract

The occurrence of popular social events causes fluctuations and changes of public emotions, while the rapid development of online social platforms and networks has made individual interactions more intense and further escalated public emotions into public opinion. However, there is a lack of consideration of individual emotions in the current research on online public opinion. Based on this, this paper firstly expounds the quantitative representation of attitude and emotion, analyzes the formation and propagation process of online public opinion by combining individual’s expression willingness, individual’s expression ability, attitude perception value, attitude change probability and other factors, and constructs a network public opinion propagation model that takes individual emotion into consideration. Finally, the main factors affecting the formation and propagation of network public opinion are discussed through simulation experiments. The results demonstrate that: (1) fear is conducive to the formation of online public opinion, but the speed is relatively slow; sadness is not conducive to the formation, but once enough people participate in the exchange of views, the formation of online public opinion will be faster; (2) the influence of online public opinion on individual emotions expands with the increase of the number of individual interactions; (3) different network structures impact differently on the propagation of public opinion. Among them, BA (BA network is a scale-free network model proposed by Barabasi and Albert in order to explain the generation mechanism of power law, BA model has two characteristics: growth and priority connection mechanism) and ER (ER network is a network with random connectivity proposed by Erdös-Renyi) random networks can promote the propagation of online public opinion, which is prone to “one-sided” online public opinion. WS small-world networks (proposed by Watts and Strogatz. It is a kind of network with short average path length and high clustering coefficient) and fully-connected networks have an inhibitory effect on the spread of online public opinion, easily maintaining the multi-dimensional nature of online public opinion.

## 1. Introduction

In a critical period of social transformation, China witnesses various contradictions and problems, leading to frequent emergencies [1]. However, the occurrence of sudden events can easily cause the fluctuation and change of public emotions. When public emotions are gradually accumulated and externalized into attitudes on such social platforms as Twitter and discussion groups, netizens’ opinions collide in propagation and discussion, thus promoting the formation of online public opinion. At the same time, online public opinion will in turn stimulate individual emotions and make individuals take part in or quit from propagation and discussion, thus affecting the propagation of public opinion. Therefore, it is of great significance to consider the propagation of online public opinion from the perspective of individual emotions. On the one hand, individual emotions will affect the formation and propagation of online public opinion. Therefore, considering the formation and propagation mechanism of online public opinion from the perspective of individual psychology is conducive to understanding public opinion and ensuring the construction of a harmonious society and the healthy development of the online environment. On the other hand, online public opinion will have an impact on individual emotions. An individual in a negative mood for a long time is likely to have various irrational behaviors, which will disturb the social order and harm the personal safety and property safety of citizens. Therefore, the online public opinion propagation model with the consideration of individual emotions is of great significance for social stability and national security.

At present, few studies on online public opinion are considered from the perspective of emotion [2]. Researchers mainly take attitude directly as an individual’s opinion on a certain event, but do not consider the generation of individual attitude and the formation of online public opinion from the perspective of psychology. Leon [3] took attitude as an individual viewpoint, studied the influence of external information on individual opinion, homogeneity of social interactions and other factors on public opinion. Studies on the relationship between individual emotions and attitudes are rarely mentioned while most researchers consider emotions are equivalent to attitudes. For example, Lee and Choi [4] explored the underlying mechanism of the effects of social viewing discussion networks on emotions in the context of the 2017 South Korean presidential debates.

In fact, emotion is an individual’s first response to an event and an internal subjective feeling, while attitude is an external expression of an individual’s views on an event, which may not really reflect the true viewpoint. Generally speaking, while being exposed to external events, individual’s inner emotions will fluctuate, and when the emotions accumulate to a certain extent, individuals may have the desire to communicate with others, promoting the externalization of individual emotions into an attitude, which is expressed in the form of language and words. Interacting with others’ opinion in social networks affects attitude changes. When the number of individuals participating in the interaction is large and over a long duration of interaction, the number of individuals holding the same attitude occupies a certain proportion, at which point the network public opinion is formed. The formation of online public opinion will have an impact on individual emotions and expression intentions, and promote the propagation of online public opinion. Therefore, from a psychological perspective, this paper integrates emotions into the formation and propagation of online public opinion. By introducing internal and external factors such as an individual’s expression willingness, expression ability, attitude perception value, attitude change probability and so on, as well as considering the formation and propagation process of network public opinion, the network public opinion propagation model considering individual emotion is constructed, so as to identify the relationship among emotion, attitude and public opinion.

The structure of this paper is as follows: Section 2 is literature review. Section 3 expounds the quantitative expression method of emotion and attitude, and models the network public opinion propagation with the consideration of individual emotion. In Section 4, the influence of individual and information characteristics and emotions on the formation of online public opinion is analyzed through simulation experiments. Meanwhile, the effects of attitude internalization, interaction times and network structure on the propagation of online public opinion are studied. Section 5 is the summary of the whole paper and the prospect of the future work.

## 2. Literature Review

At present, research on online public opinion propagation mainly focuses on three aspects: interaction mode, individual heterogeneity and network structure, while research on individual emotions mainly focuses on three aspects: emotion classification theory, emotion propagation model and online social network emotion propagation.

With regards to interaction mode, in order to understand the common influence of the majority, Das et al. [5] explicitly used the concept of opinion consistency and expertise level consistency measuring the homogeneity of a group and validated the efficacy of model in capturing opinion dynamics in real world. Li et al. [6] investigated a network stubborn individuals and orators (NSO) model based on game theory and complex social networks and set opinion guide nodes. The results show that opinion guidance is most likely to separate the public into different groups rather than converge to the guide’s opinion. In terms of individual heterogeneity, Dong and Fan [7] proposed the evolution of preferences with deceptive interactions and heterogeneous trust in bounded confidence model, unfold the influences of the deceptive interactions and heterogeneous trust on the evolution of preferences. Gastner et al. [8] introduced the concealed voter model which added a concealed layer of opinion to the public layer. If one’s public and concealed opinion disagree, an agent can reconcile them by either publicly disclosing his previously secret view or by accepting public opinion as inner conviction. Chen et al. [9,10] proposed a public opinion polarization model which integrates three categories of social preferences: egoistic, altruistic, and fair preferences and the exit rules between interactive nodes are set by revenue function, so as to expand the network from static state to dynamic state. Lee and Yan [11] proposed a model on the role of interdependent self-value and group opinion climate in affecting individuals’ willingness to participate in online political discussions. Results show that those with high interdependent self-values are more likely to engage in online civic discussions via in-depth information processing of the discussion promotion messages. In terms of network structure, based on the directivity and asymmetry of the transmission of infectious diseases, Jia et al. [12] established the SIS (SIS model is a variant of the traditional Susceptible-Infected-Recovered epidemic model. There are only two types of individuals, susceptible and infected) epidemic model in the directed Internet. The results showed that the independence between each node’s in-degrees and out-degrees significantly lowers the impact of the network’s topological structure on disease spread. Alvarez [13] created a network model with preferential attachment and found that when the network structure or the core agents in external sources support a view, the prevailing majority opinion might be promptly replaced.

Although the above researches on online public opinion propagation involve differences between individuals’ real attitudes and attitudes during interaction, most of them are explained from the perspective of individual’s preferences, without considering the causes of individual’s attitudes or differentiating individual’s inner feelings and external attitudes. In fact, when facing an emergency, an individual will feel differently. When the accumulated emotions need to be released, the individual will externalize the internal emotions into individual attitudes and interact with others with language and text as the carriers. Therefore, emotion plays an important role in the formation of individual attitude.

In terms of emotion classification theory, there are two streams, basic emotion theory and emotion dimension theory. The representative figures of basic emotion theory mainly include Levenson, Izard, Ekman, and Panksepp. They believe that the basic emotions are shared by humans and animals, with cultural commonality. Levenson [14] classified enjoyment, anger, disgust, fear, surprise, sadness into basic emotion. Izard [15] put forward six basic emotions, namely joy, sadness, disgust, fear, anger, interest. Ekman and Cordaro [16] believed there were seven basic emotions: joy, sadness, fear, anger, disgust, surprise, and contempt. Based on comparative psychology, Panksepp and Watt [17] defined basic emotions namely, seeking, fear, rage, lust, care, panic/grief, and play. Emotion dimension theory believes that emotion is not a simple existence of several basic emotions, but a highly correlated continuum, with similar emotions highly correlated on the basic dimensions. Russell [18] found that two basic dimensions of emotion (pleasure dimension and arousal dimension) can explain most of the emotional variation, and then he proposed the valence–arousal model [19], which divides basic emotion on the basis of two basic dimensions. In terms of emotion propagation, the most common mechanisms are infectious diseases and heat transfer. In 1960s, Daley and Kendal [20] found the similarity between the spread of infectious diseases and the spread of information, and proposed the classical DK model, that is, the most widely used SIR model. In 2009, Bosse et al. [21] introduced ASCIRBE model, this model is similar to the heat dissipation model in physics. The specific heat capacity of materials can be compared with the susceptibility of a person to other people’s emotions in emotional contagion. Tsai et al. [22] compared the panic propagation model based on thermodynamics and epidemiology. By simulating the ability of the two models in real crowd panic scenarios, they found that the thermodynamic model was better. In online social network, Kramer et al. [23] confirmed that emotions expressed by others on Facebook, constituting to the experimental evidence for massive-scale contagion via social networks. Fan et al. [24] imitated the way of information release in social networks, built an agent-based model, which concurrently considers emotion influence and tie strength preferences and observed anger’s preference on weak ties. By analyzing the patterns of emotional contagion with the data on Twitter, Xi et al. [25] finds that the value of transmissibility differs on different layers and on different community structures. By proposing an emotional independent cascade model, Goldenberg and Cross [26] distinguished non-digital emotion contagion and digital emotion contagion and found that emotion contagion occurred via at least three mechanisms, namely mimicry, category activation and social appraisal. Chen [27] used six emotional appraisal dimensions to evaluate four emotions, studied the impact of emotional expressions on online consumer reviews (OCRs). The results showed that OCRs with negative emotions tend to comprise more diagnostic features related the product or service, and are more informative. 

In the basic emotion theory, although the number and category of basic emotions proposed by different researchers are not the same, the classification method is basically the same. Therefore, this paper will select the four basic emotions, including joy, anger, sadness and fear, which all appear in the basic emotion theory as the research object. In addition, by integrating the emotion dimension theory, emotion will be quantitatively represented from pleasure and arousal, and subsequent simulation analysis will be carried out. As for emotional propagation, most of the above literatures regard the tweets in online social networks as the carrier of users’ emotions, believing that users can spread emotions by spreading information with emotional bias. However, the real emotions of users are not necessarily consistent with the tweets posted on social networks. Emotions are internal feeling, while the views expressed in tweets are more like the individual attitudes revealed to the public. Although some studies have mentioned that emotions affect the formation of attitudes, no further research has been conducted on the mechanism of their formation.

To sum up, in terms of online public opinion, although existing studies have focused on the differences between individual social attitudes and their real opinion, few have explained the problem from the perspective of individual psychology. With regards to emotional propagation, most researchers consider emotion is equivalent to attitude, and rarely mention the relationship between emotion and attitude. In fact, considering the source of individual opinion, inner emotions have an important influence on the formation of attitudes, which also play a significant role in the propagation of online public opinion. Therefore, there is a lack of research on the relationship between emotion, attitude and public opinion. In fact, individual attitudes and emotions are not the same. Emotion is an individual’s first reaction to an event and an inner feeling. However, the individual’s attitude towards the outside world is an externalized individual opinion, which may be influenced by the internal characteristics of the individual and the external characteristics of the event, and may not completely reflect the individual’s true views on the event. For example, when an individual’s expression intention is low due to his low externality, he will not express his attitude to others or only express part of views. As such, there is a deviation between the social attitude reflected by the group and his real ideas, which may affect public opinion monitoring. In general, when an individual is exposed to external events, his inner emotions will be mobilized. When the emotion accumulates to a certain extent, it may be externalized into an individual attitude, which promotes the formation of online public opinion. Then, it interacts with and influences other people’s attitudes in social networks to spread online public opinion. Therefore, this paper firstly discusses the relationship between emotion and attitude, defines the quantitative expression method of them. Secondly, from the two parts of network public opinion formation and propagation, combining the emotional characteristics and internal and external factors, this paper constructs the network public opinion propagation model and studies the internal mechanism of its evolution. Finally, combined with the simulation experiment, the influence of individual characteristics, information characteristics and emotions on the formation of online public opinion is studied. Meanwhile, the effect of attitude perception mode, interaction mechanism and network structure on the propagation of online public opinion is analyzed.

## 3. Model Construction

**Figure 1:**
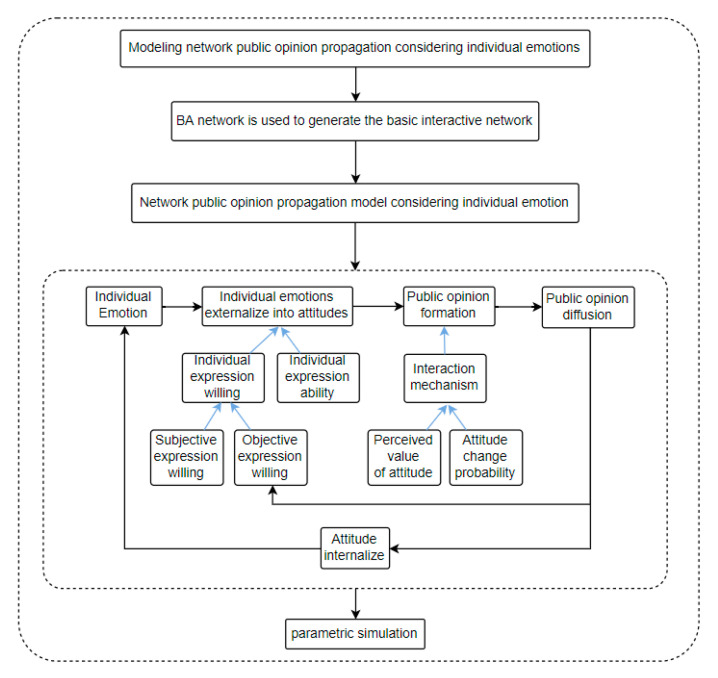
Research structure.

In this paper, modeling is carried out based on Monte Carlo multi-agent method. Agents are used to represent individual nodes in the network, and the network size is set as *N*, that is, there are *N* netizen nodes in the network. Individual emotions are expressed in a three-dimensional array, and individual attitudes are described in a two-dimensional array. The research framework of this paper is shown in Figure 1.

**Figure 2:**
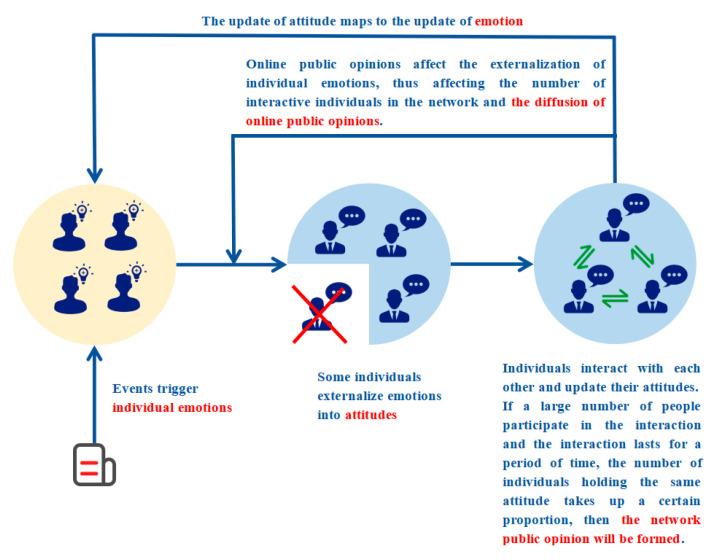
Model diagram.

According to the individual’s expression willingness and ability, when an event triggers the emotion of the Agent *i*, some individuals externalize their inner emotions into attitudes. Agent *i* uses attitude as the medium to exchange views with others and form interactions. If a large number of people participate in the interaction and the interaction lasts for a period of time, the individual attitude is stable and unchanged, and the number of individuals holding the same attitude occupies certain proportion, then the network public opinion will be formed at last. Finally, through the mechanism of attitude internalization, the change of individual attitude in the interaction process is mapped to the change of their emotion. Online public opinion also changes the number of individuals expressing their attitudes by influencing their willingness to express, that is, the number of interactive individuals, thus affecting the diffusion of online public opinion and forming a “emotion-attitude-public opinion” cycle. Based on this, the network public opinion propagation model considering individual emotions constructed in this paper is shown in Figure 2, and the main parameters and variables involved are shown in Table 1 and Table 2.

### 3.1. Quantitative Representation of Attitudes and Emotions

**Figure 3:**
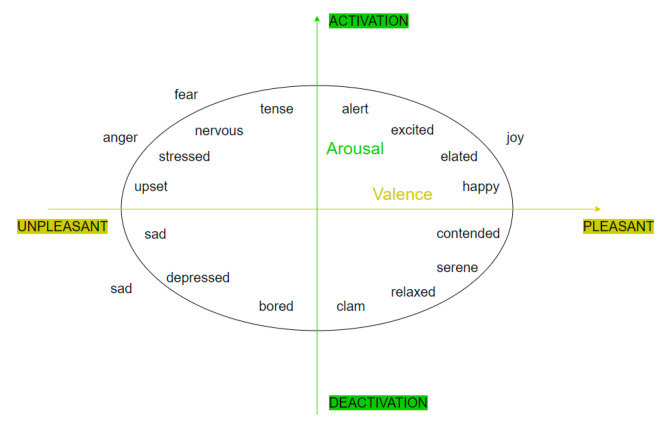
Emotional valence-arousal model.

According to the basic emotion theory [14] and the emotion dimension theory [19], this paper divides individual emotions into three dimensions. The first dimension represents the categories of basic emotions, including joy, anger, sadness and fear, which are numbered 1, 2, 3 and 4. The second dimension shows the pleasure degree, including the positive and negative emotions and emotional degree. Positivity refers to a positive or negative emotional state. The value of emotional degree is (−1, 1). The closer to −1 the value is, the stronger the negative emotion is, and the closer to 1 the value is, the stronger the positive emotion is. The third dimension is arousal degree. Emotional arousal is when you are physically or psychologically woken up or reactivated in response to external stimuli. High arousal emotions are more likely to motivate people to share and stir up emotions. As shown in Figure 3, according to the emotional valence-arousal model [19], the order of arousal degree is sadness < anger = joy < fear. In order to conduct quantitative research, this paper mapped the arousal degree of the emotional valence-arousal model into the range of (0, 1) according to its ranking, and set the arousal degree of joy, anger, sadness and fear as 0.8, 0.8, 0.6 and 1 respectively.

Considering the three dimensions of emotion, this paper uses a three-dimensional array $\left[x_{i}^{1}\left(t\right),x_{i}^{2}\left(t\right),x_{i}^{3}\left(t\right)\right]$ to represent individual emotion. Subscript *i* represents node index, superscript number represents emotional dimension, and *t* represents moment. $x_{i}^{1}\left(t\right)$ represents emotion type, $x_{i}^{2}\left(t\right)$ represents pleasure degree, $x_{i}^{3}\left(t\right)$ represents arousal degree. For example, (1, 0.8, 0.8) indicates that the emotion is a joy emotion with a positive degree of 0.8 and arousal degree of 0.8.

Although emotion is an internal experience, when it accumulates to a certain extent, it has the possibility of externalizing expression, thus affecting an individual’s attitude towards events. Due to the influence of different types of emotions, the attitude expressed by individuals also presents multi-dimensional characteristics. That is, an emotion may give rise to an attitude. For example, for the incident of “An African American being shot seven times by white policemen”, people with joy may take a schadenfreude attitude, those who have anger may denounce racial discrimination and violent law enforcement, those who have sadness may feel powerless about the living environment of black people, those who have fear may fear that they will also encounter such incidents. Therefore, individual attitude is expressed as a two-dimensional array $\left[y_{i}^{1}\left(t\right),y_{i}^{2}\left(t\right)\right]$ by referring to the quantitative expression method of emotion. Subscript *i* represents node index, superscript number represents attitude dimension, and *t* represents moment.$\;y_{i}^{1}\left(t\right)$ represents attitude type, $y_{i}^{2}\left(t\right)\;$ represents attitude degree, including positivity and attitude value. For example, (2, −0.8) represents that this attitude is the second type of attitude with negative attitude degree of 0.8. The second type of attitude refers to second type of emotion (anger), whose specific content should be based on specific events. For example, for the “African American Freud was killed” incident, the specific content of the second type of attitude is probably about racial discrimination. For “states announced to stop work” news, the specific content of the second type of attitude may be the discontent due to the lack of payment.

Individual emotions may be externalized into individual attitudes under the influence of internal and external factors, and the mapping from emotions to attitudes will be introduced in Section 3.2.1. When there is an interaction between individuals whose attitude value is not 0, their own attitudes will be updated, and the update of individual attitudes will also be synchronized with the change of individual emotions. Relevant attitudinal to emotional mapping will be introduced in Section 3.3.1.

### 3.2. The Formation of Network Public Opinion

After an event triggers individuals to produce emotion, the individual maps emotion into attitude through emotional externalization, and then forms an opinion interaction with other individuals. If the proportion of individuals participating in the interaction in the network exceeds the proportion threshold *P*_1_ formed by the network public opinion, the individual attitude tends to be stable after interaction, and the proportion of individuals holding certain attitudes exceeds the proportion threshold *P*_2_ formed by the network public opinion, then the network public opinion forms. There may be more than one attitude with a proportion higher than *P*_2_, so there may be one or more attitudes in the online public opinion.

#### 3.2.1. Emotion Externalization

After the occurrence of an emergency, with the release of initial information, individuals in the network are stimulated to generate emotions, and individuals can externalize their inner emotions into attitudes by posting tweets on social networking platforms. Generally speaking, under the influence of internal and external factors, different individuals feel differently for the same event, and their expression process is also limited by their expression ability. Based on this, combining individual expression willingness *W_i_*(*t*) and individual expression ability *δ_i_*, the mapping formula of individual emotion to attitude is shown below.

(1) $$\left(y_{i}^{1}\left(t\right),y_{i}^{2}\left(t\right)\right)=\left\{\begin{array}{c} \left(x_{i}^{1}\left(t\right),\delta_{i}\cdot x_{i}^{2}\left(t\right)\right),\;\;if\;\;W_{full}\leq W_{i}\left(t\right)\leq 1\\ \left(x_{i}^{1}\left(t\right),\frac{W_{i}\left(t\right)-W_{part}}{W_{full}-W_{part}}\cdot \delta_{i}\cdot x_{i}^{2}\left(t\right)\right),\;\;if\;\;W_{part}\leq W_{i}\left(t\right)\leq W_{full}\\ \left(0,0\right),\;\;if\;\;0\leq W_{i}\left(t\right)\leq W_{part} \end{array}\right.$$

where $y_{i}^{1}\left(t\right)\;$ and $y_{i}^{2}\left(t\right)$ are individual attitude type and individual attitude degree. $x_{i}^{1}\left(t\right)$ and $x_{i}^{2}\left(t\right)$ are individual emotion type and individual pleasure degree. *W_part_* and *W_full_* are the thresholds of individual partial and full expressions respectively. When 0 < *W_i_*(*t*) *< W_part_*, individuals refuse to express their emotions externally and hide their emotions in their hearts. Their attitude type and attitude value are both 0. When *W_part_* < *W_i_*(*t*) *< W_full_*, individuals externalize partial emotion into attitude, and the individual attitude type is consistent with the individual emotion type. When *W_full_* < *W_i_*(*t*) *<* 1, individuals externalize full emotion into attitude, and the individual attitude type is consistent with the individual emotion type.

##### Individual Expression Willingness *W_i_*(*t*)

Individual expression willingness *W_i_*(*t*) is a probability value, which is used to measure whether individual *i* will externalize emotion at time *t*. *W_i_*(*t*) belongs to (0, 1). Individual expression willingness *W_i_*(*t*) includes subjective expression willingness *WS_i_*(*t*) and objective expression willingness *WO_i_*(*t*). *WS_i_*(*t*) refers to the spontaneous expression willingness of individuals who are not affected by public opinion, while *WO_i_*(*t*) refers to the expression willingness of individuals affected by public opinion after their formation. The calculation formula of expression willingness is as follows.

(2) $$\{\begin{array}{c} W_{i}\left(t\right)=WS_{i}\left(t\right),\;\;0\leq t<t_{1}\\ W_{i}\left(t\right)=WS_{i}\left(t\right)+WO_{i}\left(t\right),\;\;t\geq t_{1} \end{array}$$

where *t*_1_ represents the moment when public opinion is formed. When *0* *≤ t* *< t_1_* (the public opinion is not formed), individuals are only affected by subjective expression willingness. When *t* *≥ t_1_* (the public opinion is formed), individuals are affected by both subjective and objective expression willingness.

The internal influencing factors of subjective expression willingness *WS_i_*(*t*)) include individual emotional characteristics and individual basic characteristics, while the external influencing factors include information characteristics. Individual emotional characteristics include pleasure degree and arousal degree. The higher the pleasure degree is, the higher the arousal degree is and the stronger the subjective expression willing is. The basic characteristics of the individual include extroversion *α_i_*. The higher the extroversion of the individual is, the stronger the subjective expression will be. Information features include life relevance *β*_1_ and political sensitivity *β*_2_. The higher the life relevance of the event is, the stronger the individual’s willingness is to express externally. The stronger the political sensitivity of the event is, the more cautious and restrained individuals are in expressing their opinion. Based on the above factors, the calculation formula of *WS_i_*(*t*) of subjective expression willingness is as follows.

(3) $$WS_{i}\left(t\right)=\left|x_{i}^{2}\left(t\right)\right|\cdot x_{i}^{3}\left(t\right)\cdot \alpha_{i}\cdot \frac{\beta_{1}}{\beta_{2}}\cdot k_{1}$$

where *k*_1_ is the regulation constant, and emotional pleasure degree $x_{i}^{2}\left(t\right)$, emotional arousal degree $x_{i}^{3}\left(t\right)$, individual extroversion *α_i_* and life relevance of information *β*_1_ are positive feedback factors, which are proportional to subjective expression willingness. Since the emotional pleasure degree of an individual can be positive and negative, its influence on the subjective expression willingness is only related to the value, and has nothing to do with the positive and negative values, so absolute value is used to eliminate the influence of positive and negative values. Political sensitivity of information *β*_2_ is a negative feedback factor, which is inversely proportional to *WS_i_*(*t*).

The formation of public opinion will have an impact on the individual’s expression willingness. For example, individual *i* feels angry towards something, but due to his low extroversion, his subjective expression willingness is low, so he does not express the attitude at the initial moment. After the formation of public opinion, the emotional bias of mainstream public opinion is anger, indicating that most individuals hold the same views as *i*, which will stimulate individual *i* to express and participate in the diffusion of public opinion. In addition, objective expression willingness is also affected by the scale of online public opinion. If more individuals express their attitudes in the network, the scale of online public opinion will be larger and the influence will be greater. Objective expression willingness *WO_i_*(*t*) represents the influence of public opinion on individual expression willingness. *WO_i_*(*t*) is calculated as follows.

(4) $$WO_{i}\left(t\right)=k_{2}\cdot \left(P_{i}\left(t\right)-0.25\right)\cdot \frac{N_{A}\left(t\right)}{N}$$

where *k*_2_ is the regulation coefficient, *k*_2_ > 0, *P_i_*(*t*) is the proportion of attitude type to be expressed by individual *i* in the public opinion field at time *t*, *N_A_*(*t*) is the number of individuals to express attitude at time *t*, and *N* is the number of netizens in the network. There are four basic emotions, corresponding to the four types of attitude, so if one particular type of attitude accounted for more than 25%, showing that the attitude in the public opinion field is in the majority, it improves the objective expression of individuals. If one particular type of attitude accounted for less than 25%, showing that the attitude in the public opinion field is in the minority, it reduces the objective expression of the individual. $\frac{N_{A}\left(t\right)}{N}$ represents that the higher the value of the proportion of people who express their attitudes online is, the greater the influence range of online public opinion and the stronger the influence on individuals’ expression will be.

**Figure 4:**
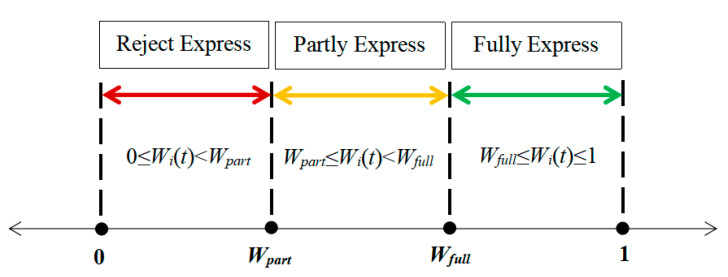
The expression range of individual emotion.

The individual’s expression willingness is divided into three sections according to the strength, with *W_part_* and *W_full_* as the critical points, as shown in Figure 4. When 0 < *W_i_*(*t*) *< W_part_*, individual expression willingness is not strong enough, and individual rejects expression. When *W_part_* < *W_i_*(*t*) *< W_full_*, the individual’s expression is relatively cautious, and his attitude is only partly expressed. When *W_full_* < *W_i_*(*t*) *<* 1, the individual’s expression is relatively open and the attitude is fully expressed.

##### Expression Ability *δ_i_*

Individual expression ability *δ_i_* affects the degree of emotion externalization. Due to people’s different social backgrounds, living environments, and learning styles, individuals’ expression abilities are all different. Generally speaking, if expression ability is stronger, the individual can convey his inner emotions more accurately, so that the attitude value expressed is close to the inner emotions. However, when the expression ability is too strong, it is possible for an individual to exaggerate his inner emotions. Supposing $\delta_{i}\in \left(0,2\right),0<{\;\delta }_{i}<1$ means that the individual with weak expression, will weaken emotion during the externalization. The closer to 1 *δ_i_* is, the more accurate the individual expression is. 1 < *δ_i_* < 2 indicates that the individual is too expressive, which will amplify the emotion during the externalization.

#### 3.2.2. Interaction of Views

When emotions are externalized into individual attitudes, individuals join in the interaction of views, and the collision of views between different individuals promotes the change of individual attitudes. In the network, individuals form a collision of views by comparing their own attitudes with others. The attitude of others perceived by individuals is influenced by internal and external factors and may be different from the attitude expressed by others. In addition, due to the influence of conformity, individuals may change their attitude types.

##### Perception Value *O_ij_*(*t*)

*O_ij_*(*t*) measures the attitude value of sender *j* perceived by receiver *i* at time *t*. The internal factors involved include the individual perception ability *λ_i_*, and the external factors include the strength of the relationship *S_ij_* and the inciting force *ε_j_*(*t*). Individual perception ability *λ_i_* and strength of the relationship *S_ij_* are set initially. *ε_j_*(*t*) is related to the emotional arousal corresponding to the attitude of the sender. The higher the emotional arousal is, the stronger inciting force it is. The calculation formula is as follows:

(5) $$\varepsilon_{j}\left(t\right)=a\cdot x_{j}^{3}\left(t\right)$$

where *a* is the adjustment coefficient and $x_{j}^{3}\left(t\right)$ represents the emotional arousal degree of sender *j* at time *t*.

According to the concept of thermodynamic model [18], with the consideration of sender, receiver and information diffusion channel, the calculation formula of attitude perception value *O_ij_*(*t*) is as follows.

(6) $$O_{ij}\left(t\right)=y_{j}^{2}\left(t\right)\cdot \varepsilon_{j}\left(t\right)\cdot \lambda_{i}\cdot S_{ij}$$

where $y_{j}^{2}\left(t\right)$ is the attitude degree of the sender *j* at time *t*, *ε_j_*(*t*) is the inciting force of the *j* at time *t*, *λ_i_* is the receiving ability of the receiver *i*, *S_ij_* is the strength of the relationship between individual *i* and *j*. The stronger the attitude value of sender *j* is, the stronger the inciting force is, the stronger the reception ability of receiver *i* is. The closer the relationship between receiver *i* and sender *j* is, the higher the attitude value perceived by individual *i* from individual *j* is.

##### The Change Probability of Attitude $T_{i}^{u}\left(t\right)$

A neighboring node refers to other network nodes directly connected with individuals, which represents users that individuals followed in social networks such as Weibo and Twitter. Facing the same event, users may express the same or different views. Therefore, in the opinion field, individual *i* is not only affected by individual neighbors with the same attitudes, but also affected by neighbor nodes with other attitudes. If the proportion of nodes with other attitudes is relatively high in neighbor nodes, and when the conformity of individuals is relatively high, the attitude types held by individuals may change. Supposing the attitude type with the highest proportion in the neighbor node of individual *i* at time *t* is *u, u* is the mainstream attitude, and the change probability of individual *i* to the mainstream attitude $T_{i}^{u}\left(t\right)$ is calculated as follows.

(7) $$T_{i}^{u}\left(t\right)=\left|Pn_{iu}\left(t\right)-Pn_{i}\left(t\right)\right|\cdot \gamma_{i}$$

where *Pn_iu_*(*t*) is the proportion of the number of neighbors with dominant attitude *u* in all neighbors of node *i* at time *t*, *Pn_i_*(*t*) is the proportion of the number of neighbors with the same attitude as node *i* at time *t* in all neighbor nodes, which are decided by the social network randomly generated at the initial moment and the emotional externalization of nodes. Since the values of the two parameters are uncertain, the difference is guaranteed to be positive through $Pn_{iu}\left(t\right)-Pn_{i}\left(t\right)$, which is proportional to the attitude change probability. *γ_i_* is the degree of individual conformity and is proportional to the attitude change probability.

##### View Interaction Mechanism

When $Pn_{i}\left(t\right)>T_{i}^{u}\left(t\right)$, the individual attitude does not change. If the attitude type *i* held by individual changes, the individual attitude value is updated with the weighted average attitude value of the neighbor node holding the mainstream attitude. If the individual attitude type does not change, the DW (DW model is a limited trust model proposed by Weisbuch and Defiuant. In this model, if two individuals have similar views, they will assimilate each other, otherwise they will not affect each other) model (is used to update its attitude value.

(1) When attitude type changes

The weighted average attitude value $y_{j^{u}}^{2}\left(t\right)$ of all neighbor nodes *j^u^* holding mainstream attitude *u* at time *t* is calculated as follows.

(8) $$y_{j^{u}}^{2}\left(t\right)=\frac{\sum_{j=1}^{N_{iu}\left(t\right)}O_{ij}\left(t\right)}{\sum_{j=1}^{N_{iu}\left(t\right)}S_{ij}}$$

where *N_iu_*(*t*) represents the number of nodes with dominant attitude *u* among neighbors of node *i* at time *t.*

If the individual’s attitude changes, $y_{j^{u}}^{2}\left(t\right)$ will be regarded as the attitude value of the next moment of the individual. At this moment, the attitude of individual *i* at *t* + 1 is calculated as follows.

(9) $$\{\begin{array}{c} y_{i}^{1}\left(t+1\right)=u\\ y_{i}^{2}\left(t+1\right)=y_{j^{u}}^{2}\left(t\right) \end{array}$$

(2) When attitude type does not change

If the attitude type held by an individual does not change, the individual attitude type at *t* + 1 is consistent with the individual attitude type at *t*, which is calculated as follows.

(10) $$y_{i}^{1}\left(t+1\right)=y_{i}^{1}\left(t\right)$$

Individual *i* interacts with all neighbor individuals who has the same attitude with *i*. The weighted average attitude value of all neighbor node *j*’ with the same attitude as individual *i* at time *t* is calculated as follows.

(11) $$y_{j^{\prime }}^{2}\left(t\right)=\frac{\sum_{j=1}^{N_{i}\left(t\right)}O_{ij}\left(t\right)}{\sum_{j=1}^{N_{i}\left(t\right)}S_{ij}}$$

where *N_i_*(*t*) represents the number of nodes with the same attitude in neighbor nodes of node *i* at time *t*.

Individuals update their attitudes through the DW model. At time *t*, individual *i* adjusts its attitude at the next moment according to the weighted average attitude value $y_{j^{\prime }}^{2}\left(t\right)$, and selects the corresponding attitude updating rules according to its difference value, as follows:

(1) When $y_{i}^{2}\left(t\right)-y_{j^{\prime }}^{2}\left(t\right)<d_{1}$,

(12) $$\{\begin{array}{c} y_{i}^{2}\left(t+1\right)=y_{i}^{2}\left(t\right)+\theta_{1}\left|y_{j^{\prime }}^{2}\left(t\right)-y_{i}^{2}\left(t\right)\right|\;\;\;\;if\;y_{i}^{2}\left(t\right)>0\\ y_{i}^{2}\left(t+1\right)=y_{i}^{2}\left(t\right)-\theta_{1}\left|y_{j^{\prime }}^{2}\left(t\right)-y_{i}^{2}\left(t\right)\right|\;\;\;\;if\;y_{i}^{2}\left(t\right)<0 \end{array}$$

where *d*_1_ is the assimilation threshold and *θ*_1_ is the assimilation parameter. When the attitude value of individual *i* is positive, *+θ*_1_ indicates enhanced attitude. When the attitude value of individual *i* is negative, *−θ*_1_ indicates reduced attitude.

(2) In other cases, the attitude value of individual *i* remains unchanged, which is expressed as follows:

(13) $$y_{i}^{2}\left(t+1\right)=y_{i}^{2}\left(t\right)$$

### 3.3. Network Public Opinion Communication

After the formation of network public opinion, public opinion will have an impact on individual emotions. At the same time, it will further affect the secondary externalization of emotions, stimulate or inhibit individuals’ expression of their own attitudes, and thus affect the communication of online public opinion.

#### 3.3.1. Attitude Internalization

In the process of interaction, individuals may be influenced by conformity and directly change their attitude types and their own attitude values, or they may be influenced by similar attitudes around them to strengthen their own attitude values. This change of attitude maps synchronously to emotions in the process, which is called attitude internalization. Attitude internalization refers to the change of an individual’s internal emotions after the change of attitude, which is related to the individual’s attitude and internalization ability at the same time. The specific calculation is as follows:

(14) $$\{\begin{array}{c} x_{i}^{1}\left(t+1\right)=y_{i}^{1}\left(t+1\right)\\ x_{i}^{2}\left(t+1\right)=\zeta_{i}\cdot y_{i}^{2}\left(t+1\right) \end{array}$$

where *ζ_i_* represents internalization ability, and $\zeta_{i}\in \left(0,\;1\right)$. After individual interactions, their attitude type corresponds to their emotional type, and their emotional value is in direct proportion to their internalization ability and attitude value.

#### 3.3.2. The secondary Expression of Emotions

After attitude internalization, the emotions of the individuals involved in the interaction change, prompting them to carry out emotional externalization again. At the same time, public opinion will also have an impact on the individuals who did not participate in the interaction. When the individuals who originally did not express emotions find that most individuals in the public opinion field hold the same views with them, they will have a stronger desire to express, so as to externalize their emotions into attitudes. Therefore, the reaction of public opinion on individuals is reflected in individual emotion and individual objective expression willingness *WO_i_*(*t*). In the process of the secondary expression of emotion, the individual has experienced the process of the externalization of emotion, the interaction of opinion and the internalization of attitude again, forming a cycle. In fact, there will be multiple cycles in the evolution of online public opinion. This paper only analyzes the communication of online public opinion by comparing the changes of the number of people participating in the interaction in the two cycles before and after, so it only discusses the secondary expression of emotions.

**Figure 5:**
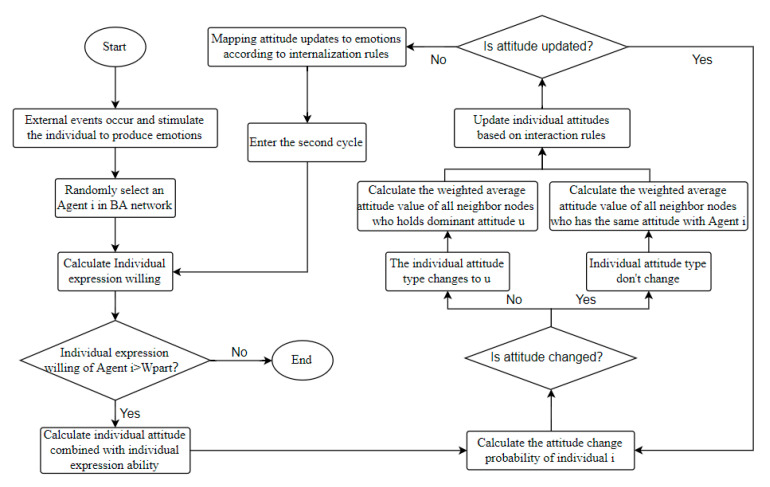
Simulation diagram based on multi-agent method proposed by Monte Carlo.

Based on the above analysis, the specific simulation process of this paper is shown in Figure 5:

## 4. Numerical Simulation Experiments

This section uses MATLAB software to simulate the formation and propagation process of network public opinion built above. This paper analyzes the impact of individual characteristics, information characteristics and emotions on the formation of online public opinion, and discusses the effect of attitude perception mode, interaction mechanism and network structure on the propagation of online public opinion, so as to reveal its internal evolution mechanism.

The initial network of the simulation experiment was using a BA scale-free network with a node size of 1000. Other parameters were set as follows: the formation parameter *P*_1_ was 50% because if *P*_1_ was too small, the number of individuals participating in the event interaction in the network was less, and the event had less influence, thus it would not attract extensive attention of netizens. Set the formation parameter *P*_2_ as 30% to ensure the formation of 1–3 kinds of network public opinion. If *P*_2_ was too small, individual attitudes would be too dispersed. If *P*_2_ was too large, individual attitude would be too concentrated. Assuming that events are highly correlated with life and political sensitivity is general, we set *β*_1_ as 0.9 and *β*_2_ as 0.5. According to the central limit theorem, people’s height, shoe size, living environment all obey normal distribution. Therefore, individual basic characteristics are set according to normal distribution. In addition, the network popularization and the development of the social platform promote individuals to participate in the discussion, so individuals also have more channels of receiving information. This article sets the extroversion of individual *α_i_*, conformity of individual *γ_i_*, receptivity of individual *ε_i_* to make all obey normal distribution of N~(0.5, 0.15). The value greater than 1 is set to 1, less than 0 is set to 0, so as to make three parameters mapping in (0, 1) interval; 0.5 represents the general receiving ability, conformity and externality of individuals in the population. Variance 0.15 is to make all the numbers within the range of (0, 1) obtain the probability value. Individual expression ability δ*_i_* is related to individual education background. Generally speaking, people with moderate expression ability occupy the majority while people with the best or poorest ability occupying the minority. Therefore, this article sets individual expression ability to obey normal distribution of N~(1, 0.2), which is mapped in [0, 2]. The average value 1 represents that 1 is the critical value. Closing to 1 is close to the true feelings, while surpassing 1 exaggerates real feelings. Variance 0.2 is to make all the numbers within the range of (0, 2) obtain the probability value. Internalization ability of individual *ζ_i_* obeys normal distribution of N~(0.9, 0.05), within the scope of mapping in (0, 1). 0.9 represents good internalization emotional abilities in the group overall. The initial states of joy, anger, sadness and fear account for about 25%, which ensures the similarity of the initial state. The initial emotions are set in positive and negative directions, and the positive emotions follow a normal distribution of N~(0.5, 0.15), which is mapped within the range of (0, 1). The negative emotion value follows the normal distribution of N~(−0.5, 0.15), and maps to the range of (−1, 0). A value of ±0.5 ensures that the initial emotions of most individuals are neutral, and very few individuals hold extreme emotions.

### 4.1. Analysis of Influencing Factors of Network Public Opinion Formation

When netizens are willing to post their opinion for some events, they will externalize their inner emotions into attitudes and participate in the interaction of views on the Internet to form public opinion. Generally speaking, if more people express their attitudes, more individuals will participate in the opinion interaction. The more nodes participate in the interaction, the more likely the network public opinion will be formed. In addition, the formation speed of online public opinion is related to the time when the interaction becomes stable. If interaction takes less time to reach a stable state, the formation of online public opinion will be faster. These two indicators representing network public opinion are not only related to individual characteristics and information characteristics, but also affected by emotions. This section will analyze these influencing factors. Except for setting *W_part_* = 0.2, *W_full_* = 0.4, *k*_1_ = 0.7, other parameters are set as shown above.

#### 4.1.1. The Influence of Individual and Information Characteristics on the Formation of Network Public Opinion

Due to the differences in education background, living environment and other factors, extroversion *α_i_* and expression ability *δ_i_* are all different, so that people have different responses to the same event. At the same time, different events have different information characteristics, leading to different reactions. Therefore, this section studies the influence of individual characteristics and information characteristics on the formation of network public opinion.

**Figure 6:**
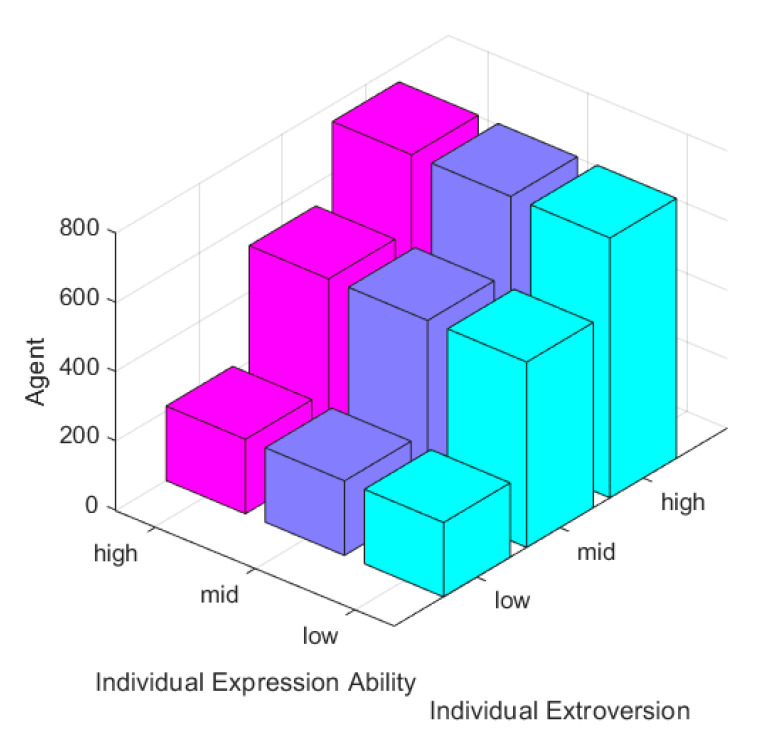
The number of people expressing their attitudes under different individuals’ extroversion and individual expression ability.

In order to describe the difference of extroversion, $\alpha_{i}\in \left(0.1,\;0.4\right)$ means low extroversion, $\alpha_{i}\in \left(0.5,\;0.4\right)$ represents the medium extroversion, and $\alpha_{i}\in \left(0.9,\;0.4\right)$ means high extroversion. All map to the range of [0, 1]. Variance 0.4 ensures that all numbers within the range of [0, 1] get probability values. The expression ability is same,$\;\delta_{i}\in \left(0.5,\;0.2\right)$ represents low expression ability, $\delta_{i}\in \left(1,\;0.2\right)$ represents medium expression ability, and $\delta_{i}\in \left(1.5,\;0.2\right)$ represents high expression ability. All map to the range of (0, 2). Variance 0.2 ensures that all numbers within the range of (0, 2) get the probability values. Based on this, the influence of individual characteristics on the formation of online public opinion is discussed, and the results are shown in Figure 6.

Figure 6 shows the number of people expressing their attitudes under different individuals’ extroversion and individual expression ability. It can be seen that higher individual’s extroversion represents more people carrying out emotional externalization. When the individuals in the group are in low extroversion degree, the number of externalized individuals is less than 500, the number of individuals participating in the interaction is too low, less than 50% of the total number of network people, and no network public opinion is formed. When all the individuals in the group are in a medium or high extroversion degree, the number of externalized individuals exceeds 500, and the number of individuals participating in the interaction is large, accounting for more than 50% of the total number of people on the network, and online public opinion can be formed. At the same time, the individual’s ability to express emotions was not related to the number of people engaged in emotional externalization. Therefore, the higher the degree of extroversion of netizen is, the more likely the formation of online public opinion is.

**Figure 7:**
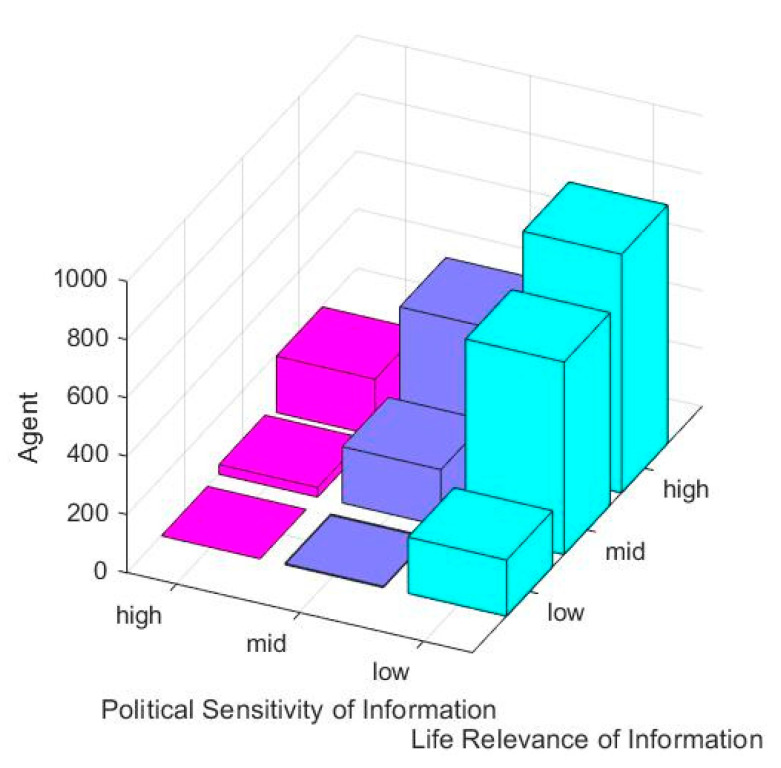
The number of people expressing their attitudes under different life relevance of information and political sensitivity of information.

In order to describe the differences of information features, *β*_1_ = 0.25 is set to represent the low life relevance. *β*_1_ = 0.5 represents the medium life relevance; *β*_1_ = 0.75 indicates high life correlation. In addition, the difference in political sensitivity is set as above. Based on this, the influence of information characteristics on the formation of network public opinion is discussed, and the results are shown in Figure 7.

Figure 7 shows the number of agents expressing their attitudes under different factors of life relevance of information and political sensitivity of information. It can be seen that the higher the life relevance of information is, the lower the political sensitivity of information is. The more individuals carrying out externalization expression are, and the more individuals participating in interaction are, the more likely network public opinion will be formed. For example, during the period of COVID-19, the discussion of COVID-19 event was higher than that of cultural events. This is because the event is closer to people’s life and less closely related to politics, therefore, people do not feel too restrained in discussion.

#### 4.1.2. The Impacts of Emotion on Public Opinion Formation

Different emotions have different degrees of arousal and pleasure, which may affect the emotional externalization of individuals and the number of nodes involved in the interaction. It may also affect the attitude perception of individuals to influence the interaction process of views. Therefore, emotion has an important influence on the formation of online public opinion. This part will study the influence of emotion on the number of nodes participating in interaction and the formation speed of online public opinion.

##### The Impacts of Emotion on the Number of Interactive Nodes

**Figure 8:**
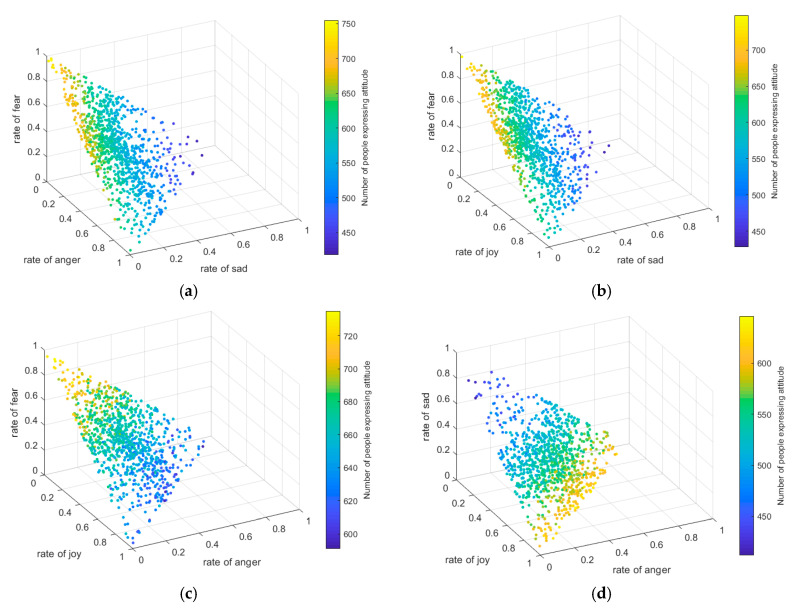
The number of individuals expressing attitude under different emotion proportion. (**a**) The number of individuals expressing attitude when rate of joy is 0, and other three emotions add to 1. (**b**) The number of individuals expressing attitude when rate of anger is 0, and other three emotions add to 1. (**c**) The number of individuals expressing attitude when rate of sadness is 0, and other three emotions add to 1. (**d**) The number of individuals expressing attitude when rate of fear is 0, and other three emotions add to 1.

The more interactive nodes make the greater influence scope of the event and the higher probability of forming network public opinion. In order to study the influence of emotion on the number of interactive nodes, the proportion of emotion in the overall network at the initial moment was randomly set, and the number of individuals expressing attitudes under different proportion of emotion was simulated, that is, the number of individuals participating in the view interaction. The results are shown in Figure 8.

Figure 8a–d shows the situation where the proportion of joy, anger, sadness and fear is 0, and the proportion of the other three emotions is set randomly. The *x*, *y, z* axes respectively represent the proportion of all kinds of emotions. The color in the figure represents the number of individuals whose attitudes have been expressed in the network. The lighter color represents the more individuals, while the darker color represents the less individuals. In order to ensure that all individuals in the network hold emotions, the sum of the other three emotions add to1.

Figure 8a,b are similar, but different from other figures, indicating that the effects of joy and anger on the number of individuals expressing attitudes are the same, while the effects of fear and sadness are different. It can be seen from Figure 8c that the color of the nodes in the figure only changed from dark to light with the increase of the value of “rate of fear” on the *z*-axis, but not with the increase of the value of “rate of joy” and the value of “rate of anger”. In addition, the same situation also appears in Figure 8d, that is, the node color only changes from light to dark with the increase of *z*-axis—“rate of sadness” value, without any response to the change of *x* and *y* axis value. This shows that when the proportion of other attitudes is constant, the proportion of joy and anger has no influence on the number of individuals expressed by the attitude, which further indicates that the effect of joy and anger is consistent. From the proportion of all kinds of emotions in the 4 figures, it can be seen that fear has the best effect of promoting emotion externalization, followed by joy/anger, and finally sadness. The cause may lie in different emotional arousal: fear arousal is easier for arousing people’s expression willingness, prompting them to share ideas with others. Sadness arousal is less able to arouse people’s expression willingness, prompting them not to express. Joy/anger arousal is moderate, so the effects are also moderate. Therefore, if the proportion of fear in the network is higher and the proportion of sadness is lower, more individuals externalize emotions into attitudes, and more individuals participate in the exchange of views, which is conducive to the formation of online public opinion.

##### The Impacts of Emotion on the Formation Speed of Public Opinion

**Figure 9:**
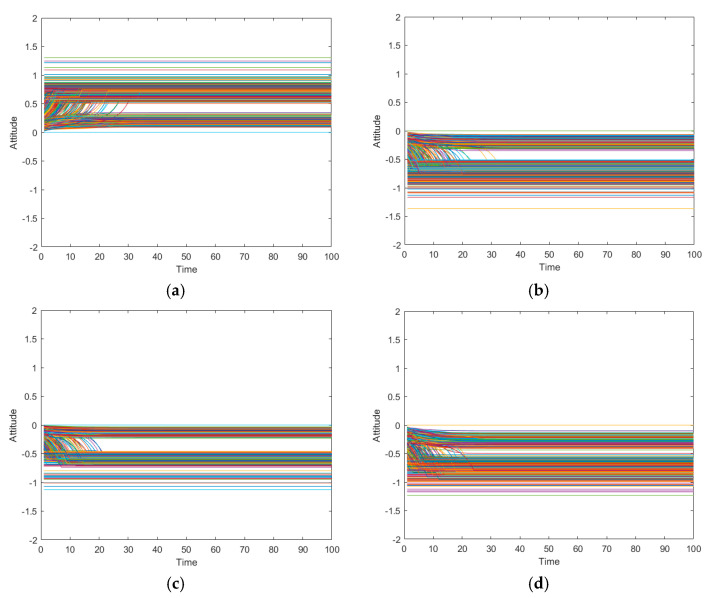
The changes of attitudes with times of interaction under different emotion proportion. (**a**) The changes of attitudes with times of interaction when joy accounts for 70%, and other three respectively accounts for 10%. (**b**) The changes of attitudes with times of interaction when anger accounts for 70%, and other three respectively accounts for 10%. (**c**) The changes of attitudes with times of interaction when sadness accounts for 70%, and other three respectively accounts for 10%. (**d**) The changes of attitudes with times of interaction when fear accounts for 70%, and other three respectively accounts for 10%.

In the viewpoint of interaction, with the increase of the times of interactions, individual attitudes may tend to be consistent and at this point, individual attitudes do not change and become stable. However, it is also possible for individuals to argue endlessly with each other, fluctuating individual attitudes constantly. In order to explore the influence of emotion on the formation speed of online public opinion, each emotion type in the network was set in proportion. The simulation results are shown in Figure 9.

Figure 9a–d show the changes of node attitudes with the number of interactions when the proportion of joy, anger, sadness and fear is 70% (70% represents the majority of individuals holding such emotions in the network). It can be seen from the above figures that when the proportion of joy and anger is relatively high, the individual’s attitude becomes stable after 30 interactions. When the proportion of sadness is relatively high, about 20 interactions are needed to reach the steady-state of individual attitude. When fear is relatively high, 20–30 interactions are needed before an individual’s attitude reaches steady state, and the results of multiple experiments are consistent. This shows that emotions have an impact on the formation speed of online public opinion. When most individuals in a group are in a sad mood, a stable state can be reached after a short period of discussion, and the formation speed of online public opinion is faster. When most of the individuals in the group are in the mood of joy and anger, the individuals need longer discussions to make their opinion more stable, and the formation time of online public opinion is slower.

The reason for this difference may be related to the number of interactive nodes and the perceived value of attitude. It can be seen from the analysis in Figure 8 that when the proportion of sadness is relatively high, the number of nodes participating in the interaction is relatively small, when the proportion of joy and anger is relatively high, the number of nodes participating in the interaction is relatively large. In real life, a team of 10 people in a conference room can reach an agreement faster than a network of thousands. Emotional arousal affects attitude perception, and the inciting force under sadness is weak, so individuals perceive others with a lower attitude value. However, the inciting force under joy and anger is stronger, and the attitude value of others perceived by individuals is higher. In real life, feelings of joy and anger are more likely to resonate with people than with sadness.

**Figure 10:**
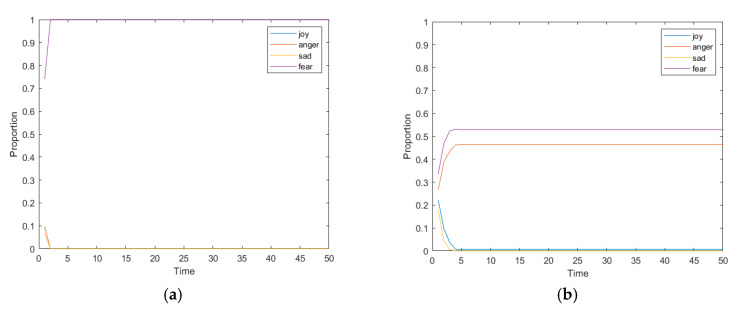
The changes of various attitudes proportion with the changes of interaction times under uneven (**a**) and even (**b**) distribution of emotion.

In order to further explore the influence of the uniformity of emotion distribution on the formation of network public opinion in the interactive process, the changes of various attitudes under the two conditions of uniform and non-uniform emotion distribution at the initial moment were simulated, and the results were shown in Figure 10.

Figure 10a shows the change of interaction times under the condition of uneven emotional distribution at the initial moment. At this point, the initial moment of fear accounted for 70%, and other three types of emotions accounted for about 10% respectively. It can be seen from the figure that, due to the uneven proportion of emotions at the initial moment, the distribution of individual attitudes at time = 1 is also very uneven. Starting from time = 2, individual attitudes change rapidly. After one interaction, all the individual attitudes in the network turn into the attitudes corresponding to fear, indicating that at this time, individual attitudes reach stable in a very short time and network public opinion with only one attitude are formed. The same result was found when the other three types of emotions were set at 70% of the initial time. Figure 10b shows the changes of the proportion of various attitudes in the network with the number of interactions under the condition of even emotional distribution at the initial moment. At this time, the proportion of the four kinds of emotions is about 25%, which is relatively averagely distributed. It can be seen from the figure that the even proportion of emotions at the initial moment also reflects the uniformity of individual attitudes in the network. At time = 1, the distribution of individual attitudes is also around 25%. It takes longer for individual attitudes to be stable, and eventually anger and fear both account for more than 30%, forming online public opinion with two attitudes. This shows that when there is no obvious bias in individual emotions triggered by events, individual attitudes are various, and the formation of online public opinion is slow. However, there may be a variety of attitudes in the network at last. However, when the individual emotional bias triggered by the event is relatively obvious, the individual attitude is affected by conformity, showing the state that the strong is stronger and the weak is weaker as in the Matthew effect. The formation of online public opinion is relatively fast, but finally presents one-side condition, and there is only one opinion in the network.

### 4.2. Analysis of Influencing Factors of Network Public Opinion Propagation

After the formation of online public opinion, the current public opinion will have a negative impact on netizens. Some individuals no longer express their attitudes or even exit from the interactive process of opinion, while some individuals choose to express their opinion and join the interactive process of opinion. The propagation of online public opinion is measured by the change in the number of individuals participating in the interaction. If the number of individuals participating in the interaction increases, the propagation of online public opinion will be promoted; otherwise, its propagation will be inhibited. Based on this, this section will study the influence of attitude internalization and interaction times on online public opinion propagation.

#### 4.2.1. The Impacts of Attitude Internalization on Public Opinion Propagation

After the formation of network public opinion, the change of individual attitude is mapped to the change of individual emotion through the process of attitude internalization, so as to affect the secondary externalization of emotion and the propagation of network public opinion. Therefore, this section analyzes the impact of attitude internalization ability *ζ_i_* on the spread of the network public opinion.

**Figure 11:**
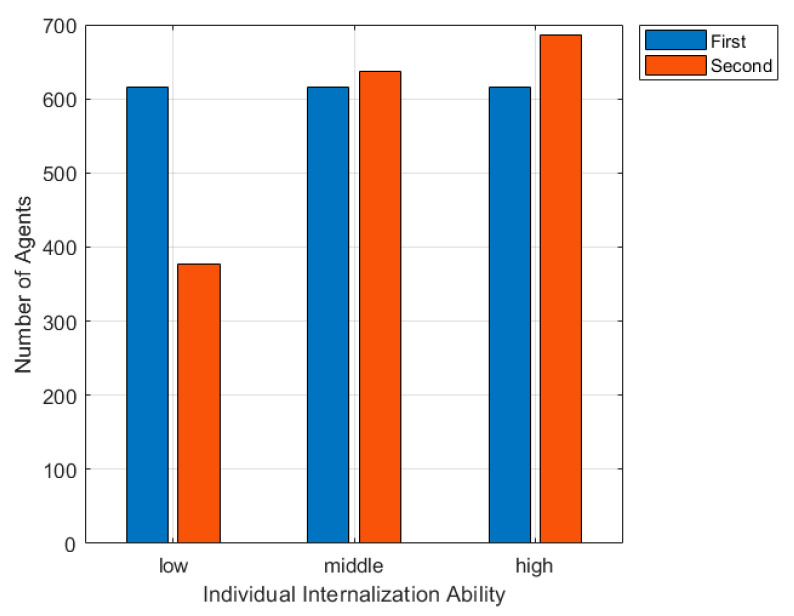
The changes of node amount participating part in interaction under different internalization abilities.

In order to describe the difference of individuals’ internalization abilities, we set $\zeta_{i}\in \left(0.9,\;0.05\right)$ as high internalization ability, $\zeta_{i}\in \left(0.5,\;0.05\right)$ as middle internalization capacity, and $\zeta_{i}\in \left(0.1,\;0.05\right)$ as low internalization ability. If *ζ_i_* < 0, it can be set as 0. If *ζ_i_* > 1, it can be set as 1. Ensuring all individual extroversion maps to (0, 1), the simulation result is shown in Figure 11.

Figure 11 is a histogram of the number of nodes participating in the interaction under different internalization abilities. As can be seen from the figure, with the continuous increase of individuals’ internalization ability, the number of individuals taking part in the viewpoint interaction through the secondary expression of emotions further increases. When individuals in a group have a weak ability to internalize, online public opinion has little impact on individual emotions, and the propagation of online public opinion is inhibited. When the internalization ability of individuals in the group is in the middle or high, the situation is on the contrary, and the propagation of online public opinion is promoted.

#### 4.2.2. The Impacts of Interaction Times on Public Opinion Propagation

During the viewpoint interaction, interaction times represent the depth of interaction. More times means deeper communication and more comprehensive understanding.

Generally speaking, the exchange of views among individuals will go through multiple stages: the change of individual views constantly stimulates the change of online public opinion, which also influences the individuals, thus forming a cycle. In order to understand the propagation of online public opinion, it is necessary to compare the changes of the number of people participating in the interaction in the two cycles. The view interaction is divided into two stages: the first emotional externalization and the second emotional externalization. The two stages are divided according to whether netizens’ attitudes are influenced by online public opinion. The first emotional externalization refers to the stage in which netizens experience emotional externalization, exchange of views and internalization of attitudes for the first time. At this stage, since the netizens are exposed to the event for the first time, whether they express opinion on the Internet depends on subjective factors only, while the interaction of opinion will promote the formation of online public opinion and ultimately lead to the change of individual emotions. The second stage of emotional externalization refers to the second stage of emotional externalization, perspective interaction and attitude internalization under the influence of public opinion after the formation of online public opinion. At this stage, whether they post an opinion on the Internet not only depends on subjective factors, but also depends on the objective factor of online public opinion, which is also related to the emotional changes caused by the previous interaction. Therefore, it is possible that either more or less individuals participate in the interaction and affect the propagation of online public opinion.

**Figure 12:**
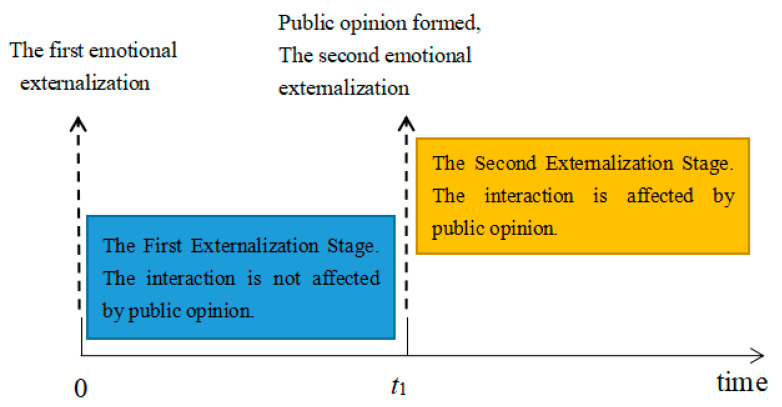
Times and stages.

Figure 12 shows the relationship between the number of interactions and the stages. Time represents the number of interactions, *t*_1_ represents the number of interactions in the first externalization stage, and it is also the time point when network public opinion affects individual interactions, which can be set independently. 0 ≤ time < *t*_1_ refers to the first stage of emotional externalization. When time = 0, individuals carry out the first emotional externalization yet following interaction of views is not affected by public opinion. When time = *t*_1_, network public opinion has been formed, and individuals carry out the second emotional externalization yet following interaction of views is affected by public opinion.

**Figure 13:**
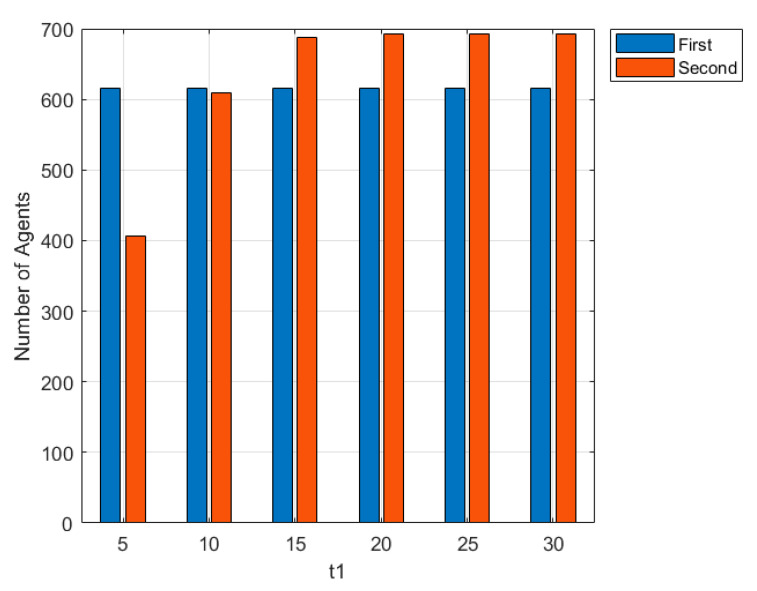
The changes of numbers of interactive nodes under different *t*_1._

**Figure 14:**
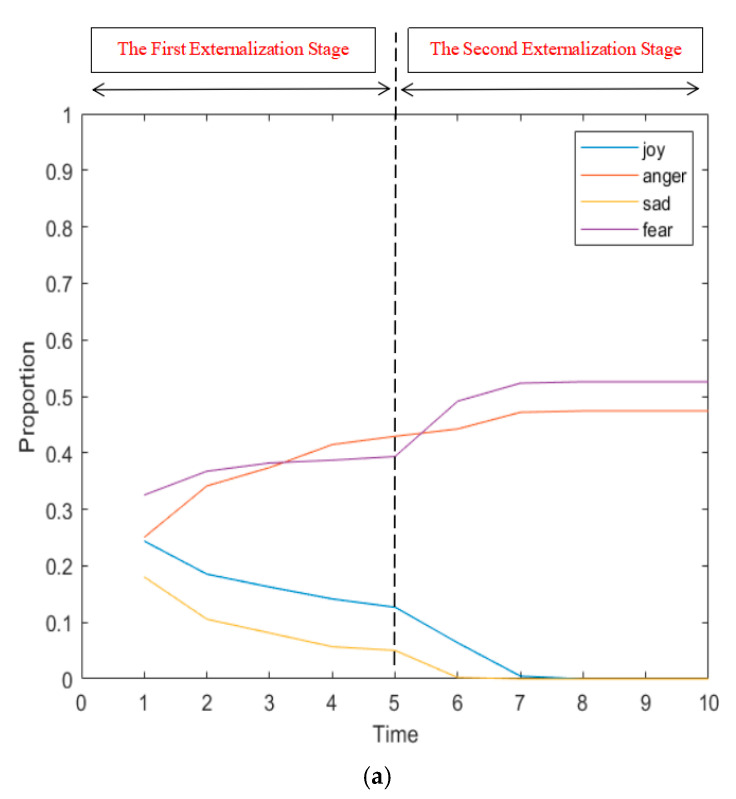

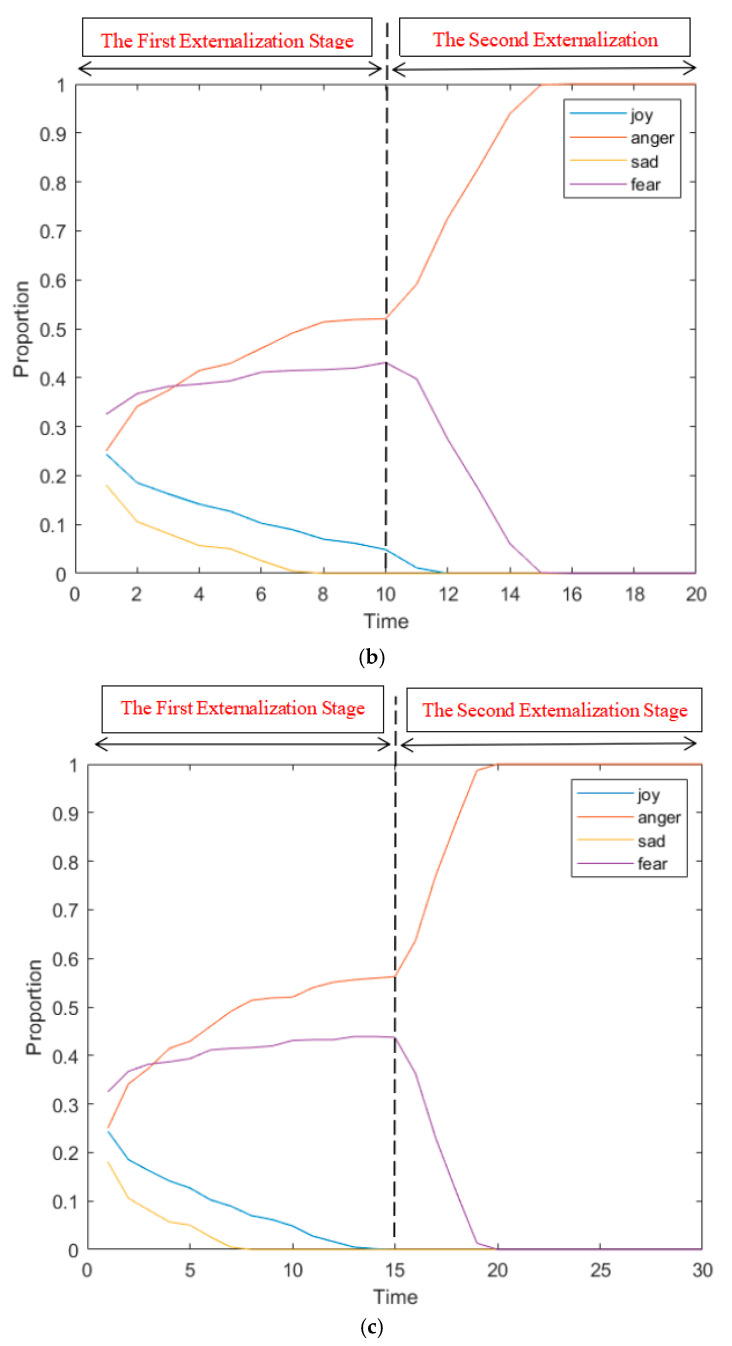
The curve of attitude proportion with the changes of interaction times under different *t*_1._ (**a**) *t*_1_ = 5, (**b**) *t*_1_ = 10, (**c**) *t*_1_ = 15.

The smaller *t*_1_ represents fewer interactions in the first emotional externalization stage and the less sufficient interactions among individuals. In order to analyze the influence of the interaction times *t*_1_ on the network public opinion propagation at the first emotional externalization stage, this part simulates the network public opinion propagation under different *t*_1_ conditions. The relevant simulation results are shown in Figure 13 and Figure 14.

Figure 13 shows the changes in the number of nodes participating in the interaction at different *t*_1_. In the first stage of emotional externalization, 615 nodes in the network expressed their attitudes and participated in the interaction. In the second stage of emotional externalization, the network public opinion formed in the previous stage were also different due to the influence of the interaction times *t*_1_ in the previous stage, which impacted on the second emotional externalization of individuals and changed the number of nodes participating in the interaction. As can be seen from the figure, when *t*_1_ = 5 and *t*_1_ = 10, the number of nodes participating in the interaction in the second emotional externalization stage was less than that in the first stage, indicating that the propagation of online public opinion was inhibited at this time. When *t*_1_ > 20, the number of nodes participating in the interaction in the second emotional externalization stage was more than that in the first stage, indicating that the propagation of online public opinion was promoted at this time. In addition, with the increase of *t*_1_, the number of nodes participating in the interaction in the second stage increases until it was maximized. FIG. 14 shows the change curve of attitude proportion with the changes of interaction times under different *t*_1_. Figure 14a–c shows the situation in which the number of individual interactions in the first stage is 5, 10 and 15, respectively. As can be seen from Figure 14a, in the first stage, when the interaction between individuals only took place for 5 times, although the proportion of attitudes in anger after the interaction was higher than that in fear, the proportion of the two attitudes changed with the interaction in the second stage of emotional externalization, and the attitude in fear slightly prevailed. As can be seen from Figure 14b,c, when *t*_1_ = 10 and *t*_1_ = 20, with the interaction in the second stage of emotional externalization, the difference in attitude proportion in the first stage is magnified. The attitude proportion in anger gets higher and higher until it occupies the whole network, and the attitude proportion in fear gets lower and lower until it is 0, and finally only one attitude exists.

The above situation shows that the number of interactions between individuals affects the propagation of online public opinion. When the interaction in the first stage of emotional externalization is not sufficient, individual opinion are scattered, and the influence of online public opinion on individual emotions is weak, and the secondary expression of emotions by individuals is inhibited to restrict the propagation of online public opinion. When the interaction in the first stage of emotional externalization is sufficient, opinion among individuals are relatively unified, and online public opinion play a strong role in individual emotions, which amplifies the differences between attitudes, promotes the secondary expression of emotions among individuals, and promotes the propagation of online public opinion. In real life, as netizens have more in-depth discussions on the event, their views on the event have become more profound and intense, and it is easier to promote other individuals to participate in the discussion.

#### 4.2.3. The Influence of Network Structure on Public Opinion Propagation

**Figure 15:**
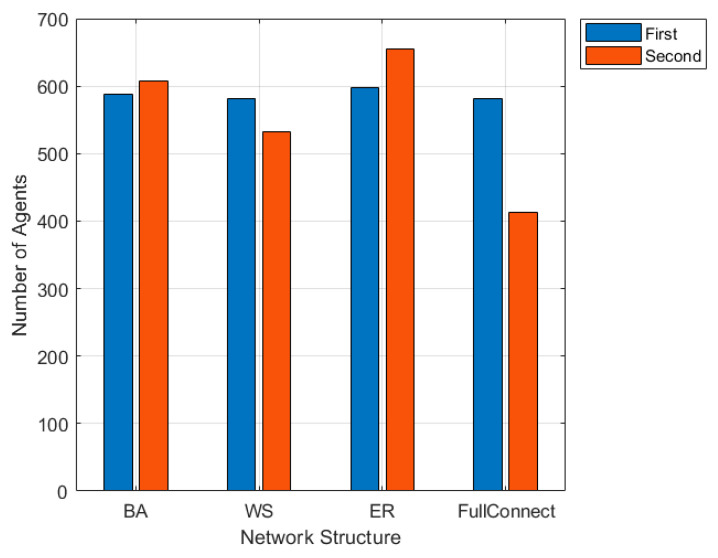
The changes of interactive nodes number under different network structure.

**Figure 16:**
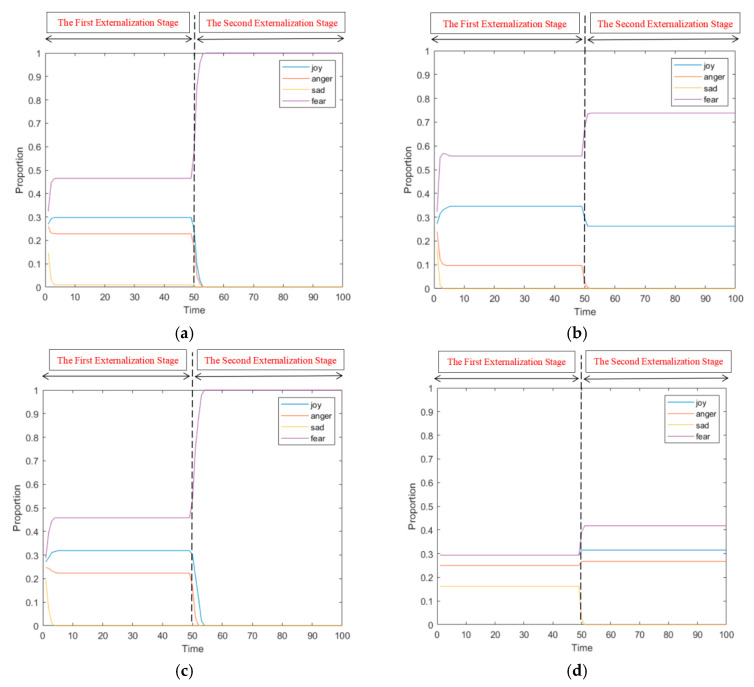
The change curve of the proportion of all kinds of attitudes in the two-stage interaction process with the number of interactions. (**a**) The change curve of the proportion of all kinds of attitudes in the two-stage interaction process with the number of interactions under BA network. (**b**) The change curve of the proportion of all kinds of attitudes in the two-stage interaction process with the number of interactions under WS small-world network. (**c**) The change curve of the proportion of all kinds of attitudes in the two-stage interaction process with the number of interactions under ER random network. (**d**) The change curve of the proportion of all kinds of attitudes in the two-stage interaction process with the number of interactions under Fully connected network.

The propagation of online public opinion is carried out in social networks. The structure of social networks has an important influence on the propagation of online public opinion. To analyze the impact of network structure of public opinion propagation model, this section chooses BA (proposed by Barabási and Albert) scale-free network [28], WS (proposed by Watts and Strogatz) small-world network [29], ER (proposed by Erdös-Renyi) random network [30] and fully connected network [31]. The average path length of a fully connected network is set to 1, and the other average path length of the network structure is similar, so as to analyze the effect of public opinion propagation under different network structures. In order to avoid the influence of interaction times on online public opinion, *t*_1_ = 50 was set to make the interaction in the first stage of emotional externalization sufficient. Different network topology parameters are shown in Table 3, and the simulation results are shown in Figure 15 and Figure 16.

Figure 15 and Figure 16 respectively show the change of the number of nodes participating in the interaction under the BA network, WS small-world network, ER random network and fully connected network, and the change curve of the proportion of all kinds of attitudes in the two-stage interaction process with the number of interactions. It can be seen from Figure 15 that the BA and ER random networks promote network public opinion propagation, while the WS small-world network and the fully connected network restrict the public opinion propagation. As can be seen from Figure 16, compared with the changes in the proportion of attitudes and the average attitude values in the BA network, the proportion and average attitude values in other network structures change slightly. Firstly, in the WS small-world network, due to the high clustering coefficient and the large number of neighbors connected by a single node, the proportion curve of attitude in the first stage fluctuates greatly and the individual attitude changes greatly. In addition, since the first stage has already interacted with more individuals and their views have been relatively objective and comprehensive, individual attitudes in the second stage change slightly, and there are two kinds of attitudes at the same time. Therefore, this network structure makes the interaction in the first stage more intense, which is conducive to the multi-dimensional development of group attitudes, but not conducive to the propagation of online public opinion. Secondly, the situation of ER small-world network is similar to that of BA network. This network structure is conducive to the spread of online public opinion, but it is easy to occur the “one-sided” situation of group attitude, so that all individuals hold the same opinion. Finally, in fully connected network, the attitude changes only when *t*_1_ = 50, which is constant in the process of interaction, and ultimately exists three types at the same time. It shows that under the network, individual contacts with neighbors with average attitudes and does not change attitude types during the interaction. However, the network public opinion will affect individual attitude. On the whole, public opinion in the four network structures have changed to different degrees, which indicates that the network structure has certain influence on the public opinion propagation.

## 5. Conclusions

Considering individual emotions, this paper constructs a network public opinion propagation model and analyzes the whole process from the formation to the propagation of network public opinion. This paper analyzes the different influences of individual characteristics, information characteristics and emotions on the formation of online public opinion, and studies the effects of attitude internalization, interaction times and network structure on the propagation of online public opinion.

The following conclusions can be drawn from the simulation experiment:

(1) Fear is conducive to the formation of online public opinion, while sadness is not conducive. However, the effect of emotion on the formation speed of online public opinion is opposite, that is, when sadness occupies the mainstream, the formation speed of online public opinion is faster. When the emotions of joy, anger and fear occupy the mainstream, the formation of online public opinion is relatively slower.
(2) The influence of online public opinion on individual emotions increases with the increase of individual interaction degree. When the interaction between individuals is not sufficient, the influence of online public opinion on individual emotions is weak, which is not conducive to the propagation of online public opinion.
(3) BA and ER random networks can promote the propagation of online public opinion, but it is easy for online public opinion to be one-sided. The WS small-world network and fully connected network have inhibitory effect on public opinion propagation, but it is easy to maintain the multi-dimensional nature of public opinion.

However, this paper still has the following deficiencies, which need further study:

(1) Since this paper involves emotional and other psychological contents, it is necessary to obtain real experimental data through ERP methodology if empirical analysis is carried out [32]. Due to the limitation of experimental conditions, this paper only makes computer numerical simulation of the model instead of empirical analysis. In addition, the empirical research of this experiment needs to conduct experiments on subjects in person. Due to the influence of COVID-19, large-scale experiments cannot be carried out temporarily. Therefore, in the follow-up research, further research can be conducted in the empirical aspect.
(2) The propagation of online public opinion is usually a dynamic process. Although this paper sets the increase and decrease mechanism of network nodes through objective expression intention, it does not take some situations into account, such as individuals’ different online time and forgetting degree in real social networks. Therefore, in the follow-up research, it is necessary to fully consider the increase and decrease mechanism of nodes in the network in combination with real social networks [33].

## Figures and Tables

**Table 1:** Parameters involved in the model.

Symbol	Meaning	The Model Used
*P*_1_	The threshold of interactive proportion that affects the formation of network public opinion	The formation of network public opinion
*P*_2_	The threshold of attitude proportion that affects the formation of network public opinion	
*α_i_*	Extroversion of individual *i*	Emotion Externalization
*δ_i_*	Expression ability of individual *i*	
*β*_1_	Life relevance of event information	
*β*_2_	The political sensitivity of the event information	
*W_part_*	The threshold of partial expression of attitude	
*W_full_*	The threshold of full expression of attitude	
*γ_i_*	The conformity of individual *i*	Interaction of views
*λ_i_*	Receptivity of individual *i*	
*d*_1_	The assimilation threshold of DW model	
*ζ_i_*	Internalization ability of individual *i*	Attitude internalization

DW model is a limited trust model proposed by Weisbuch and Defiuant. In this model, if two individuals have similar views, they will assimilate each other, otherwise they will not affect each other.

**Table 2:** Variables involved in the model.

Symbol	Meaning	The Model Used
$x_{i}^{1}\left(t\right)$	The emotion type of node *i* at time *t*	All
$x_{i}^{2}\left(t\right)$	The emotional value of node *i* at time *t*	
$x_{i}^{3}\left(t\right)$	Emotional arousal value of node *i* at time *t*	
$y_{i}^{1}\left(t\right)$	Attitude types of node *i* at time *t*	
$y_{i}^{2}\left(t\right)$	Attitude value of node *i* at time *t*	
*W_i_*(*t*)	Individual expression willing of node *i* at time *t*	Emotion Externalization
*WS_i_*(*t*)	Subjective expression willing of node *i* at time *t*	
*WO_i_*(*t*)	Objective expression willing of node *i* at time *t*	
*P_i_*(*t*)	The proportion of attitude type to be expressed by node *i* in public opinion field at time *t*	Interaction of views
*N*	Number of network nodes	
*N_A_*(*t*)	The number of people expressing attitudes in the network at time *t*	
*N_iu_*(*t*)	The number of nodes with dominant attitude *u* among neighbors of node *i* at time *t*	
*N_i_*(*t*)	The number of nodes with the same attitude in neighbor nodes of node *i* at time *t*	
*O_ij_*(*t*)	Perception value of attitude of node *i* to node *j* at time *t*	
*S_ij_*	The strength of the relationship between node *i* and node *j*	
*ε_j_*(*t*)	Inciting force of node *j*	
*Pn_iu_*(*t*)	The proportion of the number of neighbors with dominant attitude *u* in all neighbors of node *i* at time *t*	
*Pn_i_*(*t*)	The proportion of the number of neighbors with the same attitude as node *i* at time *t* in all neighbor nodes	
$T_{i}^{u}\left(t\right)$	The change probability of node *i*’s attitude changing into dominant attitude *u* at time *t*	
$y_{j^{\prime }}^{2}\left(t\right)$	The weighted average attitude value of all neighbors *j*’ of node *i* who has the same attitude as node *i* at time *t*	
$y_{j^{u}}^{2}\left(t\right)$	The weighted average attitude value of all neighbors *j^u^* of node *i* with dominant attitude *u* at time *t*	

**Table 3:** Different network topology parameters.

Name	Average Path Length	Clustering Coefficient	Average Degree
BA scale free network	2.5742	0.0558	19.3200
WS small-world network	2.6405	0.3756	30
ER random network	2.6327	0.0198	20.2260
Fully connected network	1	1	999
